# Measures of Maternal Metabolic Health as Predictors of Severely Low Milk Production

**DOI:** 10.1089/bfm.2021.0292

**Published:** 2022-07-12

**Authors:** Laurie A. Nommsen-Rivers, Erin A. Wagner, Dayna M. Roznowski, Sarah W. Riddle, Laura P. Ward, Amy Thompson

**Affiliations:** ^1^ Department of Rehabilitation, Exercise, and Nutrition, University of Cincinnati College of Allied Health Sciences, Cincinnati, Ohio, USA.; ^2^ Cincinnati Children's Hospital Medical Center, Cincinnati, Ohio, USA.; ^3^ Department of Obstetrics and Gynecology, University of Cincinnati College of Medicine, Cincinnati, Ohio, USA.

**Keywords:** insufficient milk, human lactation, low milk supply, metabolic health, insulin resistance, metabolic syndrome

## Abstract

**Background:**

A comprehensive approach to breastfeeding support requires elucidation of how metabolic health influences milk production.

**Objective:**

We compared metabolic health indicators in women with severely low milk output versus those with moderate/normal milk output using a case–control study design, with nested and external control groups.

**Design:**

Cases and nested controls were derived from women screened for a low milk supply trial, with cases defined as severely low milk output (<300 mL/24 hours), and nested controls defined as moderate/normal milk output (>300 mL/24 hours). In addition, we included an external control group of exclusively breastfeeding women. All were enrolled at 2–10 weeks postdelivery of a healthy term infant. Milk output and breast emptying frequency were recorded through test-weigh. Metabolic health variables included all components of the metabolic syndrome, homeostatic model assessment of insulin resistance (HOMA-IR), and diagnosis of gestational diabetes mellitus (GDM).

**Results:**

Maximum milk output, mL/24 hours, ranged as follows: 30–281 in cases (*n* = 18), 372–801 in nested controls (*n* = 12), and 661–915 in external controls (*n* = 12). Mean breast emptying frequency in cases was not significantly different from nested or external controls. All metabolic syndrome components and HOMA-IR were significantly worse in cases as compared with both nested and external control groups (*p* < 0.05). There was no significant difference between the nested and external control groups for these variables. GDM prevalence was 39%, 0%, and 8%, across cases, nested control, and external control groups, respectively (chi-square *p*-value = 0.02).

**Conclusion:**

Results from this small case–control study identify class 2+ obesity and poor metabolic health as strong risk factors for severely low milk production. These findings should be further validated in larger prospective cohort studies designed to identify individuals at risk for metabolically driven low milk supply. In addition, clinical and qualitative research studies aimed at improving patient-centered approaches to the management of persistent low milk supply are needed.

## Introduction

Exclusive breastfeeding to 6 months, with continued breastfeeding to 1 year or longer, is strongly endorsed by multiple public health agencies.^1,2^ More than 8 of every 10 U.S. mothers initiate breastfeeding, but only half will meet their own breastfeeding goals, with low milk supply being a top reason for breastfeeding discontinuation.^3,4^ It is a long-held belief that nearly all mothers are physiologically capable of producing adequate breast milk.^5,6^ However, this truism may not apply to women with obesity^7^ or diabetes.^8^ Maternal obesity and diabetes are consistent risk factors for delayed lactogenesis.^9–12^ and shortened breastfeeding duration.^13–16^ Early bovine^17^ and rodent research^18,19^ demonstrates that excess body fat impedes milk production, but consensus is lacking as to why obesity and diabetes increase the risk of delayed lactogenesis and shortened breastfeeding duration^20^

Women with obesity may have lower prolactin response to suckling in the first few days postpartum,^21^ but the clinical implications of this finding, including its impact on sustained lactation, are unknown. Breastfeeding interventions targeting women with obesity (peer support^22^ and breast pumping^23^) have not been effective in improving breastfeeding outcomes. Within the context of the current global obesity epidemic, there is a critical need to identify the maternal metabolic health indicators that are most strongly associated with insufficient milk production, beyond the broad risk association with body mass index (BMI).

Previously, we conducted a pilot randomized controlled trial (RCT) of metformin to increase milk production in women with signs of insulin resistance.^24^ We measured metabolic parameters and 24-hour milk output as part of screening for trial eligibility. Improvement in milk output in the metformin versus placebo groups was small (+64 mL/24 hours) and not statistically significant in this pilot trial. However, among all women screened at baseline, we did observe significantly lower milk output in women with general signs of insulin resistance (gestational diabetes mellitus [GDM], polycystic ovary syndrome, abdominal obesity, or elevated fasting plasma glucose) compared with women with none of these signs.^24^

Our objective here is to compare a comprehensive set of metabolic health parameters in women with severely low milk output with those with moderate/normal milk output among women screened for trial eligibility at baseline, with additional comparison with a separate external control group of exclusively breastfeeding women. We hypothesize that women with severely low milk output will have significantly worse metabolic health across several measures, as compared with women from the same study with higher milk production (nested controls), as well as compared with women in an exclusively breastfeeding external control group.

## Subjects and Methods

### Participants enrolled in baseline screening for the pilot RCT

Between February 2015 and June 2016, local lactation support clinicians referred mothers who desired to exclusively breastfeed but whose infants required supplementation due to insufficient milk production. Some mothers self-referred. We screened mothers through telephone for these initial inclusion criteria: 1–8 weeks postpartum and ≥20 years of age, with a healthy singleton infant born at ≥37 weeks gestation; and exclusion criteria: breastfeeding and/or breast pumping <6 times total per side/24 hours based on maternal report, unwilling to continue frequent breast emptying for 2–4 weeks, living outside the catchment area, lack of established pediatric care for infant, history of breast surgery, current maternal diagnosis of type 1 or type 2 diabetes, current nipple or breast infection, or current metformin use.

**FIG. 1.**
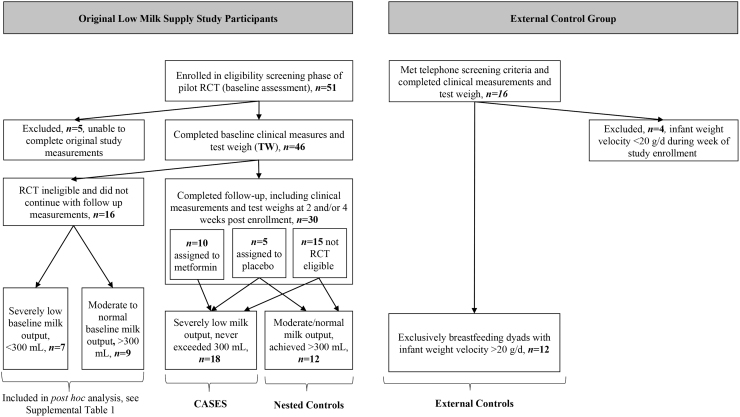
Derivation of severely low milk output cases, nested control group, and external control group. Cases include all 10 assigned to metformin and 4 assigned to placebo in the original RCT, plus 4 nonrandomized low milk supply study participants. RCT, randomized controlled trial.

We telephone-screened 114 women over 18 months of recruitment. Of the 51 who met telephone screening criteria, 46 completed clinical screening measurements (baseline), of whom 30 completed follow-up measurements 2–4 weeks postbaseline, including 10 who were randomized to metformin, 5 who were randomized to placebo, and 15 who were not eligible or declined RCT participation. Hereafter, we collectively refer to these participants as the low milk supply study participants (Fig. 1).

### Participants enrolled in the exclusively breastfeeding external control group

From March through June of 2018, we recruited exclusively breastfeeding mothers for a study of hourly milk production rate^25^ through communication with in-person parent support groups and parenting social media sites. We screened potential participants through telephone for these inclusion criteria: 4–10 weeks postpartum at time of screening (to capture a postpartum timeframe comparable with the pilot RCT follow-up measurements), mother ≥20 years of age, and currently exclusively breastfeeding a healthy singleton infant born at ≥37 weeks gestation; and these relevant exclusion criteria: living outside the catchment area, history of breast surgery, current maternal diagnosis of type 1 or type 2 diabetes, evidence of insufficient infant weight gain based on maternal report of infant weight history,^26^ or tandem breastfeeding.

Consenting mothers were further screened for inclusion in the exclusively breastfeeding external control group based on completion of the test-weigh procedure and our direct measurement confirming that their exclusively breastfeeding infant was exhibiting appropriate weight velocity, which we defined as gaining at least 20 g/day over the week of study measurements.^26^ Thus, mothers in the exclusively breastfeeding external control group were enrolled under selection criteria similar to mothers enrolled in the baseline assessment phase of the pilot RCT, with the notable difference being that they were successfully exclusively breastfeeding infants with documented adequate weight gain. Hereafter, we refer to these participants as the external control group (Fig. 1).

### Measurement of maternal milk output and infant milk intake

For both the low milk supply study participants and the external control group, we conducted a home visit to instruct the participant on proper test-weigh technique for the measurement of milk output. For the low milk supply study participants, an International Board-Certified Lactation Consultant provided a comprehensive breastfeeding evaluation and guidance in stimulating milk production with frequent and thorough breast emptying through breastfeeding and breast expression before providing test-weigh instruction. For some mothers (particularly those who self-referred), identification of ineffective or infrequent breast emptying at this home visit improved the participant's low milk supply problems and concerns. In these situations, the participant's baseline test-weigh data often reflected normal or near-normal milk output.

We provided participants in both studies with a Tanita digital infant scale (±2g; Tanita, Inc., Arlington Heights, Illinois) to use during the study and employed the “teach-back” method to ensure participant understanding of the test-weigh technique. The participant then completed at-home infant test-weighing, which included continuous recording of the weight of the infant or breast milk collection container before and after every breastfeeding, bottle feeding, or breast milk expression.^27^ Low milk supply study participants were instructed to continue with test-weighing for 24 consecutive hours, whereas the external control group was instructed to continue with test-weighing for 48 consecutive hours.

Our rationale for the longer timeframe for the external control group was that in normal lactation there is day-to-day variation in milk output as measured by infant test-weighing due to variation in infant appetite. Conversely, low milk supply study participants were instructed to frequently empty their breasts above and beyond infant breastfeeding; thus, 24 hours of test-weighing represented their current milk output capacity.

We promptly reviewed the completed test-weigh record for each participant and assessed the record for accuracy and biological plausibility. We considered odd-numbered entries as suspect (because the study scales only record even numbers in the weight range of the study infants), and we also considered suspect entries reflecting >200 g gain or >2 g loss in breast milk transfer from a single breast. In these situations, we asked the participant to repeat or extend the test-weigh timeframe.

Our goal was to calculate milk output capacity in the low milk supply study participants, and breast milk intake for exclusively breastfeeding infants with healthy weight gain in the external control group. To determine maternal milk output per 24 hours for the low milk supply study participants, we summed milk output for each breast starting from the end time of the first test-weigh entry (i.e., starting with an empty breast) to the end time of the last test-weigh entry for that breast (i.e., ending with an empty breast), then corrected to g/24 hours and summed for both breasts. For the external control group, we summed infant intake (breastfeeding and any feeding of expressed breast milk) starting with the first test-weigh entry (i.e., starting with a hungry infant) and ending with the start time of the last test-weight entry (i.e., ending with a hungry infant) then corrected to g/24 hours.

Breast emptying frequency was derived from counting the number of breastfeeds and breast emptying episodes for each breast during the test-weigh time frame, adjusting to 24 hours and summed for both breasts. Thus, if an infant fed on both sides at a “feeding” this counted as two breast emptying episodes; similarly, if the mother used a double breast shield set to collect breast milk from both breasts at a single pumping session, this counted as two breast emptying episodes.

### Assessment of metabolic health variables

For the current analysis, we included variables that comprise the metabolic syndrome risk score or metabolic syndrome criteria, measures of insulin resistance, and perinatal health conditions. These measures include BMI, waist circumference, blood pressure, fasting plasma glucose, insulin, c-peptide, and lipids, serum prolactin, GDM diagnosis in the index pregnancy, and polycystic ovary syndrome. All participants came to the Cincinnati Children's Schubert Research Clinic within 1 week of completing the test-weigh instruction home visit. Participants arrived at the research clinic in the morning after an overnight fast.

A research clinic nurse measured blood pressure in duplicate.^28^ The nurse then collected two fasting blood samples 5 minutes apart, and our research staff oversaw the immediate processing and timely transfer of the blood samples to Cincinnati Children's Clinical Laboratory. Plasma samples were assayed within 30 minutes for fasting plasma glucose, triglycerides, and cholesterol concentrations using enzymatic colorimetric methods performed on automated equipment. Aliquots of serum from the two fasting blood draws were combined and stored at −80°C for submission to Cincinnati Children's Biochemistry Core for batch assay of c-peptide, insulin, and prolactin through chemiluminescence immunoassay based on the sandwich principle.^29^

For the external control group, this blood sample was obtained at about 2 hours after the last breastfeeding/breast emptying episode, an additional blood sample was obtained 30 minutes after the initiation of breastfeeding, and these samples were assayed for basal and postbreastfeeding prolactin concentrations, respectively. For the low milk supply study participants, we were not able to control for the timing of breastfeeding or breast expression relative to the timing of the fasting blood draw; therefore, prolactin results coded as ‘basal’ if the blood sample was taken at least 90 minutes after the last breast emptying episode, and as postbreastfeeding if the blood sample was taken <90 minutes from the start of the most recent breastfeeding.

Research staff interviewed participants regarding demographics, medical history, and breastfeeding history. Cincinnati Children's Nutrition Core conducted anthropometric measurements in duplicate to obtain maternal height (±0.1 cm), weight (±0.2 kg), and mid-waist circumference (±0.1 cm). Anthropometry was repeated if >0.1 cm discrepancy for height or circumference, or >0.2 kg discrepancy for weight. We derived naked weight by subtracting estimated clothing weight using a list of preweighed apparel.

### Low milk supply study follow-up measurements

Most participants in the low milk supply study also provided follow-up measurements of milk output and metabolic health (either as part of the pilot RCT or as optional observational participation only). For these participants, we repeated the test-weigh procedure and research clinic measurements at 2 weeks (optional) and 4 weeks after the baseline measurements.

## Ethics

We followed procedures on human research participation in accordance with the ethical standards of the Institutional Review Boards of Cincinnati Children's Hospital Medical Center (for the low milk supply study participants) and the University of Cincinnati (for the external control group). Study participants provided written informed consent before study protocol initiation. Whenever we identified infants with weight gain <20 g/day, a study pediatrician followed up with the mother. This follow-up always included sending written documentation to the infant's pediatrician and, as appropriate, may have included a recommendation to breastfeed more frequently, or for follow-up care for breastfeeding assessment and support.

## Statistics

### Derived variables

We calculated BMI as kg/m^2^. We used fasting glucose, fasting triglycerides, fasting HDL cholesterol, systolic blood pressure, waist circumference, and maternal race/ethnicity to calculate metabolic syndrome risk *z*-score, which is calculated from an algorithm where 0, >0, and <0 *z*-scores signify average, worse than average, and better than average cardiometabolic health, respectively, relative to all U.S. adults aged 20–64 years.^30^ We also calculated the homeostatic model assessment of insulin resistance (HOMA-IR) using the formula: (fasting c-peptide [ng/mL/mL] × fasting glucose [mmol/L])/22.5.^31^ We used c-peptide rather than insulin in calculating HOMA-IR, because it is a more stable measure of insulin resistance.^32^

We divided the low milk supply cohort into two groups based on whether their maximum measured milk output was above or below 50% of 600 mL, which is the lower range of normal milk intake of exclusively breastfeeding infants.^33,34^ We designated these groups as the moderate/normal output nested control group (≥300 mL) and severely low output cases (<300 mL).

For the primary analysis we restricted the low milk supply records to those with follow-up test-weigh data, as we were able to prospectively assess if low milk supply was persistent in these records. For this analysis we used clinical data from the baseline assessment and used the highest recorded milk output value (whether it was at the baseline test-weigh, or after 2 or 4 weeks of follow-up) to classify their milk output group. None of the participants who were assigned to metformin in the original RCT ever achieved milk output >300 mL. Secondarily, we repeated the analysis with the full sample of low milk supply participants, using the baseline test-weigh to classify milk output group for those without follow-up data.

We conducted data analysis using SAS 9.4 for Windows (Cary, NC). In these analyses, we summarized participants' characteristics as mean (standard deviation [SD]) for continuous variables and as frequencies (%) for categorical variables, stratified by output group (external control group, moderately low/normal nested control group, and severely low cases group). We log-transformed all hormone measurements before data analysis. We used analysis of covariance with Tukey–Kramer post hoc multiple comparison test to identify significant differences in mean values among the three groups and Fisher's exact test to examine differences in frequencies among the three groups. For select metabolic health variables, we conducted receiver-operator curve (ROC) analysis to identify the predicted cutoff with optimal sensitivity (Se) and specificity (Sp) in identifying cases of severely low milk output.^35^ For all analyses we defined significance as *p*-value <0.05.

## Results

Of the 16 participants initially eligible for the external control group, 4 of their infants did not meet our minimum cutoff for weight gain velocity (weight gain was <20 g/day during their week of study participation). Evaluation of the latter cases by the study lactation consultant identified inadequate or irregular feeding frequency and poor milk transfer at the breast.^25^ Thus, 12 participants with complete test-weighs and who were exclusively breastfeeding infants with healthy rates of weight gain comprised the external control group. Their infants' breast milk intake ranged from 661 to 915 mL/24 hours at 28–65 days of age.

Of the 46 participants in the low milk supply cohort with baseline measurements, 30 also had follow-up test-weigh measurements. Of the latter 30, the maximum measured milk output in 12 nested controls was >300 mL, ranging from 372 to 801 mL/24 hours, over a follow-up period that began at 13–57 days postpartum and continued to 40–93 days postpartum; and in 18 cases the maximum measured milk output was <300 mL, ranging from 30 to 281 mL/24 h, over a follow-up period that began at 7–61 days postpartum and continued to 21–101 days postpartum (Fig. 1).

Table 1 presents a comparison of demographic characteristics, lactation variables, and metabolic health variables across the milk output groups described in the previous paragraph. Mothers were similar in demographic characteristics across groups, except for the severely low cases having a lower prevalence of graduating from college (*p* = 0.05). Among the lactation variables, the timing of the baseline test-weigh for the nested control group and severely low output cases was about 2 weeks earlier than the average postpartum day of the external control group test-weigh measurements, but the final and maximum milk output time points were not significantly different from the external control group.

**Table 1.** Characteristics by Lactation Group, Low Milk Supply Cohort Restricted to Those Who Completed the Follow-Up Test-Weigh Measurements

	External control group,* n* = 12	Low milk supply cohort		^a^p
		Moderate/normal milk output nested controls (≥300 mL),* n* = 12	Severely low milk output cases (<300 mL),* n* = 18	
	Mean (SD) or Median [Q1–Q3] or %			
Maternal and infant characteristics				
Maternal age, years	32 (4)	34 (4)	33 (6)	0.66
College graduate	92%	100%	67%	0.05
Primiparous	33%	50%	56%	0.54
Vaginal delivery	83%	75%	50%	0.18
Female infant	67%	50%	50%	0.73
Newborn weight loss ≥10%	0%	75%	83%	0.0001
Lactation variables				
Milk output at baseline, g/24 hours	758 (71) a^b^	539 (166) b	162 (73) c	<0.0001
Breast emptying events at baseline	14 (4) a	25 (8) b	19 (6) ab	0.001
Postpartum day started baseline test-weigh	46 (13) a	31 (14) b	28 (17) b	0.003
Milk output increased, %	n/a	58%	50%	0.72
Maximum milk output, g/24 hours	758 (71)^c^ a	604 (151) b	183 (72) c	<0.0001
Breast emptying events at maximum output	14 (4)^c^	20 (8)	17 (4)	0.06
Postpartum day of maximum output	46 (13)^c^	53 (22)	40 (18)	0.18
Postpartum day of final test-weigh	46 (13)^c^	65 (18)	55 (20)	0.20
Max milk output ≥600 g/24 hours, %	100%	58%	0%	<0.0001
Metabolic health variables				
Postpartum day of baseline lab measurements	51 (13) a	35 (14) b	30 (17) b	0.003
BMI, kg/m^2^	26.2 (6.6) a	28.4 (4.2) a	38.7 (8.3) b	<0.0001
BMI category				
Normal	58%	25%	6%	0.003
Overweight	25%	42%	11%	
Obese				
I (30.0–34.9)	0%	25%	17%	
II (35.0–39.9)	8%	8%	28%	
III (≥40.0)	8%	0%	39%	
^d^Waist circumference, cm	88.9 (12.3) a	93.3 (10.1) a	112.0 (15.5) b	<0.0001
^d^Fasting plasma glucose, mg/dL	85 (6) a	85 (5) a	92 (8) b	0.007
Fasting insulin, U/mL	4.6 [3.8–8.5] a	6.8 [4.3–8.1] ab	9.0 [5.9–17.1] b	0.004
Fasting c-peptide, ng/mL	1.38 [1.10–2.09] a	1.63 [1.22–1.84] a	2.36 [1.96–2.95] b	0.0007
HOMA-IR_C-peptide_	0.29 [0.22–0.47] a	0.34 [0.24–0.41] a	0.50 [0.43–0.74] b	0.0006
^d^Plasma triglyceride, mg/dL	63 (29) a	68 (15) a	124 (60) b	0.0005
^d^HDL cholesterol, mg/dL	70 (15) a	61 (10) ab	55 (13) b	0.01
LDL cholesterol, mg/dL	98 (28)	110 (33)	120 (39)	0.24
Total cholesterol, mg/dL	181 (34)	184 (33)	199 (44)	0.38
^d^Systolic blood pressure, mm	103 (10) a	105 (10) a	117 (10) b	0.0008
Diastolic blood pressure, mm	67 (7) a	69 (9) a	80 (9) b	0.0001
Metabolic syndrome risk *z*-score	−0.99 (0.67) a	−0.68 (0.43) a	+0.39 (0.73) b	<0.0001
Serum prolactin, basal, uIU/mL	1,193 [757–1,655] *n* = 12	2,028 [1,524–2,734] *n* = 6	677 [463–2,007] *n* = 12	0.07
Serum prolactin post breastfeeding, uIU/mL	4,071 [2,267–7,989] *n* = 12	3,199 [2,504–8,539] *n* = 6	3,351 [1,972–7,324] *n* = 6	0.92
Gestational diabetes mellitus	8%	0%	39%	0.02
Polycystic ovary syndrome	17%	8%	22%	0.87

Notes: milk output, g/24 hours, is based on exclusively breastfed infant intake for external control group and on total milk output for the low milk supply cohort. Breast emptying events/24 hours is the sum of left breastfeeds + left breast expression sessions + right breastfeeds + right breast expression sessions, normalized to 24 hours. For the low milk supply cohort, prolactin was categorized as “basal” if the single blood draw was obtained at least 1.5 hours after the most recent breast emptying episode and categorized as “response” if obtained <90 minutes from the start of the most recent breast emptying episode. For the external control group, “basal” was obtained at least 2 hours after a breast emptying episode and “response” was obtained 30 minutes after the start of the most recent breast emptying episode. To convert prolactin values to ng/mL, divide by 21.2.

^a^ *p*-Value based on ANOVA for continuous variables or log-transformed continuous hormone variables, and Fisher's exact test for categorical variables.

^b^ Differing online letters denote significantly different means (*p* < 0.05) based on ANOVA post hoc Tukey–Kramer test.

^c^ Baseline values repeated to enable statistical comparison with follow-up time points in the low milk supply cohort.

^d^ This variable is a component of the metabolic syndrome risk *z*-score, which is an algorithm where 0, >0, and <0 *z*-scores signify average, worse than average, and better than average metabolic health profiles, respectively, as compared with all U.S. adults aged 20–65 years.

ANOVA, analysis of variance; BMI, body mass index; HDL, high-density lipoprotein; HOMA-IR, homeostatic model assessment of insulin resistance; LDL, low-density lipoprotein; SD, standard deviation.

Mean (SD) [range] follow-up output changed +31 (121) [−172 to 256] mL and −7 (65) [−149 to 118] mL from baseline in the nested control group and severely low output cases, respectively, *p* = 0.34. Among the seven (58%) from the nested control group who increased milk output, the average increase was 112 (70) mL, and among the nine (50%) from the severely low output cases who increased milk output, the average increase was 42 (38) mL. In the nested control group, breast emptying frequency was significantly higher than in the external control group at baseline, but not at the time of maximum milk output.

Mean (SD) timing of the blood sample relative to the most recent breastfeeding or breast pumping was 2.8 (1.4) hours and 2.6 (0.7) hours for the basal prolactin measurement and was 41 (26) and 13 (7) minutes for the prolactin response measurement, in the nested control group and cases, respectively. Although not statistically significant, median basal prolactin was substantially lower in the severely low output cases; however, no participants had basal prolactin levels <300 μIU/mL (∼15 ng/mL) or response prolactin <600 μIU/mL (∼30 ng/mL).

Among the metabolic health variables, BMI, waist circumference, glucose, c-peptide, HOMA-IR, triglycerides, systolic blood pressure, diastolic blood pressure, and metabolic syndrome risk *z*-score were all significantly different in the severely low milk output cases as compared with both the external control group and the nested control group. For these same variables, there was no significant difference between the nested control group and the external control group. GDM prevalence was also significantly different across groups.

For the aforementioned 10 metabolic health variables, we conducted ROC analysis to determine area under the curve and estimate optimal cutoffs for identifying cases of severely low milk output. Given that we did not observe any substantial or statistically significant differences in these same metabolic health variables between the nested control group and external control group, we combined these two controls groups for ROC analysis (*n* = 24 controls). We further confirmed this step by using *t*-tests to compare metabolic variables within the nested group, split at average maximum milk output (604 mL), and found no substantial or statistically significant difference (data not shown).

Also, given that GDM status will almost always be known, we included this variable in parallel with each metabolic health variable in our ROC models. With the “in parallel” approach, if either the cutoff or GDM status is met, the record is considered test-positive.^35^ ROC analysis results are presented in Table 2. The largest area under the ROC curve was for metabolic syndrome severity *z*-score, followed by BMI, then waist circumference. The least useful ROC curves were for glucose and GDM, but even for these latter two variables the 95% confidence interval was >0.50, indicating better predictive power than chance (Table 2).

**Table 2.** Receiver Operator Curve Results for Select Metabolic Health Variables in Parallel with Gestational Diabetes Mellitus in Identifying Mothers with Persistent Severely Low Milk Output (<300 mL/24 Hours)

Metabolic health parameter	AUC (95% CI) for model that includes GDM	Se, true positive fraction at optimal cutoff	Sp, true negative fraction at optimal cutoff	Optimal Se/Sp cutoff in parallel with GDM	Metabolic syndrome cutoff^a^
Metabolic syndrome severity *z*-score^b^	0.92 (0.83–1.00)	0.89 (16/18)	0.92 (22/24)	≥0.30	n/a
BMI	0.88 (0.77–0.99)	0.94 (17/18)	0.83 (20/24)	≥33.3 kg/m^2^	n/a
Waist circumference	0.89 (0.79–0.99)	0.89 (16/18)	0.79 (19/24)	≥104 cm	≥89 cm
Diastolic blood pressure	0.85 (0.74–0.97)	0.89 (16/18)	0.71 (17/24)	≥75 mm Hg	≥85 mm Hg
Systolic blood pressure	0.83 (0.70–0.96)	0.94 (17/18)	0.75 (18/24)	≥110 mm Hg	≥130 mm Hg
Plasma triglycerides	0.83 (0.71–0.95)	0.72 (13/18)	0.92 (22/24)	≥130 mg/dL	≥150 mg/dL
Plasma c-peptide	0.83 (0.70–0.95)	0.83 (15/18)	0.75 (18/24)	≥2.0 ng/mL	n/a
HOMA-IR_C-peptide_	0.82 (0.72–0.95)	0.78 (14/18)	0.83 (20/24)	≥0.5	n/a
Plasma glucose	0.77 (0.62–0.92)	0.83 (15/18)	0.75 (18/24)	≥90 mg/dL	≥100 mg/dL
GDM	0.67 (0.55–0.80)	0.39 (7/18)	0.96 (23/24)	Positive diagnosis	n/a

Notes: Data are combined for the external control group and the moderate/normal nested control group (*n* = 24 total) and contrasted against the severely low milk output cases (*n* = 18). Optimal Se and Sp cutoffs were derived from models that combined each metabolic health parameter with GDM, where test positive is defined as either the cutoff or GDM being present (i.e., applying two diagnostic criteria in parallel). Results are ordered from highest AUC to lowest, based on the trapezoid rule and optimal Se/Sp is based on the shortest distance formula (SAS v9.4).

^a^ Metabolic syndrome cutoffs are based on the criteria used at NIHLB (https://www.nhlbi.nih.gov/health-topics/metabolic-syndrome).

^b^ Metabolic syndrome severity *z*-score, which is an algorithm where 0, >0, and <0 *z*-scores signify average, worse than average, and better than average metabolic health profiles, respectively, as compared with all U.S. adults aged 20–65 years.

AUC, area under the curve; CI, confidence interval; GDM, gestational diabetes mellitus; n/a, not applicable; Se, sensitivity; Sp, specificity.

**FIG. 2.**
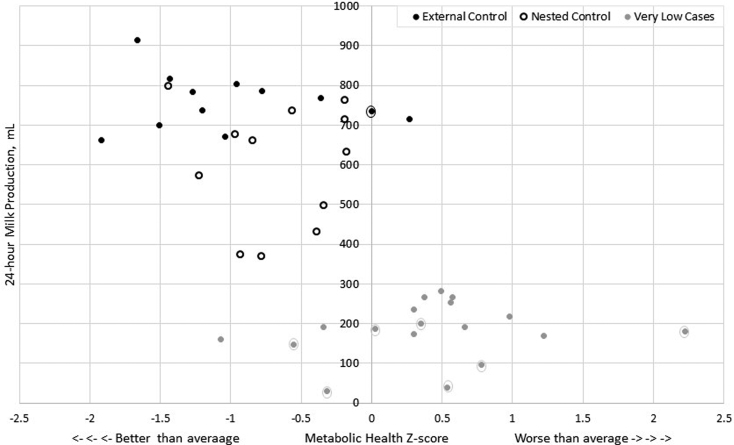
Scatterplot of 24-hour milk production by Metabolic Syndrome Severity *z*-score, where 0, > 0, and < 0 *z*-scores signify average, worse than average, and better than average metabolic health profiles, respectively, as compared to all U.S. adults aged 20–65. *Solid black circles*: external control group, *n* = 12; *Open black circles*: nested control group, *n* = 12; *Solid gray circles*: severely low milk output cases, *n* = 18. Encircled markers of any color indicate gestational diabetes mellitus diagnosis, *n* = 7 cases and *n* = 1 external control. GDM, gestational diabetes mellitus.

Figure 2 presents a scatterplot of milk output by metabolic syndrome risk *z*-score, with GDM status and milk output group labeled for each data point (*n* = 42).

Supplementary Table S1 presents a comparison of the same variables as in Table 1, but for the full sample of original participants in the low milk supply study (*n* = 58), irrespective of whether there was a follow-up test-weigh to confirm persistence in their baseline status. As shown in Figure 1, inclusion of these participants adds 7 cases and 9 nested controls to the comparison. The results in Supplementary Table S1 are highly consistent with the results in Table 1.

## Discussion

Our analysis is the first to our knowledge to directly assess metabolic health parameters in women with persistent severely low milk production. We were able to mitigate possible control group selection bias by comparing metabolic health parameters in the cases to both a nested control group of women enrolled into the same study and using the same selection criteria as the cases, but with moderate/normal milk output, and to an external control group of exclusively breastfeeding mothers of infants with healthy weight gain.

The nested control group was quite heterogenous, as evidenced by about 50% persisting with a moderate deficit in milk production despite normal metabolic health (Fig. 2) and sustained effort at regular breast emptying over 2–4 weeks of follow-up, pointing to the multiple etiologies of insufficient milk production. Although there are many possible causes of insufficient milk production, our results suggest that poor metabolic health could be an important yet understudied contributor to persistent severely low milk production.

Because the timing of metabolic health measurements was significantly later for the external control group as compared with the low milk supply cohort, we ran paired *t*-tests in the latter (*n* = 30) to determine if any of the metabolic health measurements changed significantly between baseline and follow-up time points, which were an average of 29 ± 4 days apart. Overall, there was little change. For example, on average, BMI increased 0.1 kg/m^2^ from baseline to follow-up (*p* = 0.44) and metabolic syndrome risk *z*-score increased 0.04 units from baseline to follow-up (*p* = 0.46). The only two measurements that did change significantly were low-density lipoprotein (LDL) and total cholesterol (about 10 mg/dL decline for both).

These results are very similar to Lovelady, where LDL and total cholesterol declined significantly between 6 and 12 weeks postpartum in the control group of an intervention study in lactating women, but there was not a significant decline in fasting glucose or insulin.^36^ The dynamic nature of early postpartum cholesterol may detract from its value as a marker of metabolic health in lactation. However, several other biomarkers were highly predictive in differentiating those with severely low milk production from the nested controls and the external control group, all being strongly associated with insulin resistance and inflammation,^30^ yet little research to date examines how these factors influence human lactation.

A physiological hallmark of obesity is insulin resistance.^37^ For decades, prevailing theory discounted a *direct* role for insulin in lactation.^38,39^ Beginning in 2009, in vitro studies challenged this theory, demonstrating a direct and essential role for insulin in lactation.^40–43^ In addition, research demonstrates that insulin stimulates milk protein and lipid biosynthesis in human lactation.^44^ The first author and collaborators have consistently reported associations between glucose intolerance and negative lactation outcomes in human studies.^8,10,45^

Our results support that GDM is a significant risk factor for insufficient milk production. In our study, GDM prevalence was 8% in the external control group versus 39% in the severely low milk output cases. This finding is consistent with an earlier case–control study that we conducted using data from a breastfeeding medicine clinic^8^ in which we reported 6% prevalence of diabetes in pregnancy in controls (women diagnosed with latch or nipple problems, but without low milk supply), versus 15% in cases (women diagnosed with low milk supply without latch or nipple problems).

Previously the large prospective cohort SWIFT study reported that greater lactation intensity and duration is protective against the development of type 2 diabetes in women who were diagnosed with GDM, leading to the conclusion that women with GDM should be encouraged to exclusively breastfeed.^46^ However, our results suggest that reverse causation may underlie at least part of the reported results. Specifically, the same women who were unable to achieve exclusive breastfeeding may have been more metabolically vulnerable to development of type 2 diabetes, as posited by Stuebe.^47^

Our results are the first to our knowledge to link elevated triglycerides and blood pressure with severely low milk production. However, both are part of the constellation of factors that are driven by excess visceral adipose stores and inflammation, increasing the risk of developing cardiovascular disease and type 2 diabetes.^30^ Inflammation is shown to impair lactation in rodent models.^48^ Translating to humans, an analysis of a large perinatal cohort reported significantly higher odds of stopping breastfeeding within the first month postpartum in women with a higher inflammatory diet pattern during pregnancy.^49^

Elevated plasma triglycerides are also associated with suboptimal liver function and dysregulation of lipoprotein lipase, and it is recently postulated that these factors play key role in successful lactation.^50^ As part of the low milk supply study, we isolated lactocyte mRNA from fresh milk, and in a subsequent report we will probe mammary gene expression signatures that may reveal how inflammation, lipid metabolism dysregulation, and insulin resistance contribute to severely impaired milk production.

One limitation of our study is our inability to include potential upstream influences on lactation outcomes. For example, the influences of metabolic health during adolescence on pubertal breast development and subsequent clinical implications remain largely unknown and certainly deserve greater attention.^51^ Also, we were unable to control for differences in breastfeeding management before study enrollment. However, at the time of study observation, the mothers in the low milk supply study were emptying their breasts at least as frequently as the external control group, if not more so.

In contrast, the overall conclusion is strengthened by the finding that the metabolic profile of the severely low group was significantly different than both the nested control group and the external control group. This supports the hypothesis that despite potential pubertal influences or possible suboptimal early breastfeeding management, there is an additional strong insult on milk production that is associated with current poor metabolic health. Furthermore, we had the opportunity to confirm persistent insufficient milk production despite documented continued frequent breast emptying over 2–4 weeks of follow-up.

Another limitation of our study is the inadequate evidence base for evaluating perinatal metabolic health, which gave us no choice but to use standards established for the general population of adults. To identify pregnant and lactating people who are metabolically at risk for insufficient milk production, there is a critical need for research to establish healthy normal ranges that are aligned to gestational or postpartum week.

A final limitation of our study is the relatively small sample size; nonetheless, it reports novel findings using measured milk production combined with strong characterization of metabolic health captured during the small window of time that the participants were actively invested in increasing milk production.

Even though case–control studies have their limitations, one advantage is their efficiency in examining a novel hypothesis with a relatively small sample size.^52^ It would likely require a prospective cohort study of several hundred lactating women enrolled at birth and followed carefully over the first 4–8 weeks postpartum to confirm the same number of severely low milk supply cases as we have obtained from our low milk supply study. Despite the small sample sizes, the differences in metabolic health between cases and control groups were large enough to be statistically significant with very small *p*-values.

To further elucidate the role of metabolic health, and to establish reliable cutoffs for identifying women at high risk for severely low milk production, it will be necessary to enroll a large longitudinal cohort starting in pregnancy, with particular focus on measures of metabolic health and mammary development. Furthermore, the fact that we observed only small improvements in milk output (at best), despite sustained regular breast emptying, points to the need for patient-centered outcomes research toward a more viable approach to management of persistent low milk supply.^53^ In the meantime, our novel results suggest that it would be prudent for breastfeeding mothers with class II obesity or higher (>35 kg/m2), and/or with GDM, to be prioritized for closer follow-up of breastfeeding progress and newborn weight change after maternity unit discharge.

## Supplementary Material

Supplemental data
