# Supplementary material for: Measures of Maternal Metabolic Health as Predictors of Severely Low Milk Production
Source: Breastfeed Med. 2022 Jul 12;17(7):566–76. doi: 10.1089/bfm.2021.0292 (PMC9299530; doi:10.1089/bfm.2021.0292)
Supplement: Supplemental data [file Supp_TableS1.docx]

**Supplemental Table 1.** Characteristics by lactation group, with sample size maximized

|  | External Control Group, *n*=12 | Low Milk Supply Cohort | | ^1^*p*-value |
| --- | --- | --- | --- | --- |
|  |  | Moderate/Normal Milk Output Nested Controls (>300 mL), *n*=21 | Severely Low Milk Output Cases  (<300 mL), *n*=25 |  |
|  | Mean (SD) or Geometric Mean [Q1-Q3] or % | | |  |
| ***Maternal and infant characteristics*** | | | | |
| Maternal age, years | 32 (4) | 33 (4) | 32 (6) | 0.90 |
| College graduate | 92% | 86% | 64% | 0.09 |
| Primiparous | 33% | 43% | 56% | 0.40 |
| Vaginal delivery | 83% | 76% | 52% | 0.09 |
| Female infant | 67% | 52% | 52% | 0.67 |
| Newborn weight loss >10% | 0% | 67% | 76% | 0.48 |
| ***Lactation variables*** | | | | |
| Milk output/24 h at timepoint of maximum output | 758 (71)  a^2^ | 534 (176)  b | 169 (87)  c | <0.0001 |
| Breast emptying /24 h at maximum output | 14 (4)  a | 19 (7)  b | 18 (6)  ab | 0.08 |
| Postpartum day of maximum milk output | 45 (13)  a | 42 (22)  b | 37 (18)  b | 0.41 |
| Max milk output > 600 mL/24 h, % | 100% | 38% | 0% | <0.0001 |
| ***Metabolic health variables (measured within 1-2 days of baseline lactation measurements)*** | | | | |
| Postpartum day of clinical measurements | 51 (13)  a | 34 (15)  b | 31 (17)  b | 0.002 |
| BMI, kg/m^2^ | 26.2 (6.6)  a | 29.3 (5.5)  a | 36.7 (9.5)  b | 0.0003 |
| BMI Class  Normal  Overweight  Obesity  I (30.0-34.9)  II (35.0-39.9)  III (>=40.0) | 58%  25%  0%  8%  8% | 24%  33%  29%  10%  5% | 12%  12%  20%  20%  36% | 0.009 |
| ^3^Waist circumference, cm | 88.9 (12.3)  a | 91.4 (11.9)  a | 106.0 (20.1)  b | 0.003 |
| ^3^Fasting plasma glucose, mg/dL | 85 (6)  ab | 84 (5)  a | 90 (8)  b | 0.005 |
| Fasting insulin, U/mL | 4.6 [3.8 – 8.5]  a | 6.3 [3.9 – 7.8]  a | 8.0 [5.9 – 16.3]  b | 0.002 |
| Fasting C-peptide, ng/mL | 1.43 [1.10 -2.09]  a | 1.63 [1.16 -1.77]  a | 2.13 [1.87 – 2.84]  b | 0.0002 |
| HOMA-IR_C-peptide_ | 0.30 [0.22-0.47]  a | 0.34 [0.23 – 0.37]  a | 0.49 [0.40 – 0.64]  b | 0.0002 |
| ^3^Plasma triglyceride, mg/dL | 63 (29)  a | 70 (15)  a | 134 (87)  b | 0.0005 |
| ^3^HDL cholesterol, mg/dL | 70 (15)  a | 63 (10)  a | 53 (14)  b | 0.0009 |
| LDL cholesterol, mg/dL | 98 (28) | 114 (36) | 123 (37) | 0.12 |
| Total cholesterol, mg/dL | 181 (34) | 191 (39) | 202 (41) | 0.29 |
| ^3^Systolic blood pressure, mm Hg | 103 (10)  a | 110 (10)  ab | 115 (9)  b | 0.005 |
| Diastolic blood pressure, mm Hg | 67 (7)  a | 71 (9)  ab | 77 (10)  b | 0.005 |
| Metabolic syndrome risk z-score | -0.99 (0.67)  a | -0.71 (0.46)  a | +0.24 (0.90)  b | <0.0001 |
| Gestational diabetes mellitus | 8% | 5% | 28% | 0.08 |
| Polycystic ovary syndrome | 17% | 5% | 28% | 0.12 |
| Serum prolactin, basal, uIU/L | 1193 [757 -1655]  n=12  ab | 1633 [1068 -2162]  n=13  a | 559 [380 – 1370]  n=16  b | 0.02 |
| Basal prolactin < 300 uIU/mL | 0% | 0% | 6% | 1.0 |
| Serum prolactin post breastfeeding, uIU/mL | 3944 [2267 – 7989]  n=12 | 3791 [2539- 7174]  n=8 | 3680 [1972-4843]  n=9 | 0.70 |
| Prolactin post breastfeed <600 uIU/mL | 0% | 0% | 0% | -- |

^1^*p*-value based on ANOVA for continuous variables or log-transformed continuous hormone variables, and Fisher’s Exact Test for categorical variables.

^2^Differing letters denote significantly different means (*p*<0.05) based on ANOVA *post-hoc* Tukey-Kramer test.

^3^This variable is a component of the Metabolic Syndrome Risk Z-score, which is an algorithm where 0, <0, and >0 z-scores signify average, worse than average, and better than average metabolic health profiles, respectively, as compared to all U.S. adults aged 20-65.

*Notes:* For the low milk supply cohort, **sample size was maximized** whereby all participants who completed the baseline screening are included. For those with follow-up test weigh data, their maximum milk output was used for analysis, otherwise, milk output is based on baseline. **Milk output, g/24 hours,** is based on exclusively breastfed infant intake for external control group and on total milk output for the low milk supply cohort. **Breast emptying events/24 hours,** is the sum of left breastfeeds + left breast expression sessions + right breastfeeds + right breast expression sessions, normalized to 24 hours. For the low milk supply cohort, **prolactin** was categorized as ‘basal’ if the single blood draw was obtained at least 1.5 hours after the most recent breast emptying episode and categorized as ‘response’ if obtained less than 90 minutes from the start of the most recent breast emptying episode. For the external control group, ‘basal’ was obtained at least 2 hours after a breast emptying episode and ‘response’ was obtained 30 minutes after the start of the most recent breast emptying episode. To convert prolactin values to ng/mL, divide by 21.2.
